# Bailout Percutaneous Balloon Mitral Valvuloplasty for Bioprosthetic Mitral Valve Stenosis as a Bridge to Surgery

**DOI:** 10.1016/j.jaccas.2025.105416

**Published:** 2025-09-17

**Authors:** Mahmoud Shehta, Youssef AbdelMoneim, Rana Ayman AbdelHamid, Fady Medhat Nessim, Hani El Galab, Sameh S. Raafat

**Affiliations:** a Department of Cardiology, Ain Shams University Hospitals, Cairo, Egypt; b Department of Anesthesiology and Intensive Care Medicine, Ain Shams University Hospitals, Cairo, Egypt; c Department of Cardiothoracic Surgery, Ain Shams University Hospitals, Cairo, Egypt

**Keywords:** mitral valve, rheumatic heart disease, valve replacement

## Abstract

**Background:**

Percutaneous mitral valvuloplasty is the treatment of choice for clinically significant native mitral valve stenosis, however there have been limited reports of successful balloon valvuloplasty for bioprosthetic mitral valve stenosis.

**Case Summary:**

A 44-year-old woman presented with decompensated heart failure and severe bioprosthetic mitral valve stenosis. She developed cardiogenic shock and was deemed at prohibitive operative risk. Urgent percutaneous balloon valvuloplasty was performed, resulting in hemodynamic stabilization and improvement of mitral valve area to 1.6 cm^2^.

**Discussion:**

A literature search identified 16 cases of balloon valvuloplasty of bioprosthesis degeneration in the mitral position; nearly all patients, as in our case, experienced symptom improvement.

**Take-Home Message:**

Percutaneous balloon valvuloplasty for bioprosthetic mitral valve degeneration and stenosis can be a valuable salvage strategy in highly selected patients with severe hemodynamic compromise as a bridge to definitive surgical treatment.

## Graphical Abstract

**Figure:**
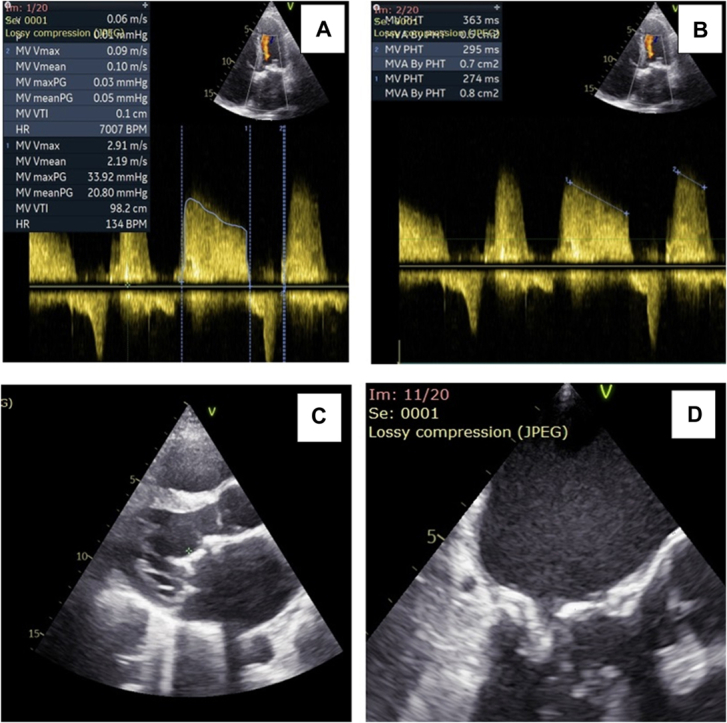

Bioprosthetic mitral valve degeneration and stenosis is a challenging late complication after mitral valve replacement. Although redo surgery remains the standard treatment, many patients present with prohibitive surgical risk owing to hemodynamic instability or comorbidities. Transcatheter valve-in-valve implantation has emerged as an alternative therapy, but anatomical and logistic limitations may preclude its use. In such circumstances, percutaneous balloon mitral valvuloplasty may serve as a bridge to definitive treatment. We present a case of a hemodynamically unstable patient with severe stenosis of a degenerated bioprosthetic mitral valve that was successfully managed with urgent percutaneous balloon valvuloplasty as a bridge to redo surgery.

## History of Presentation

A 44-year-old woman presented with progressive dyspnea on exertion, assessed as NYHA functional class III, accompanied by symptoms of right-sided heart failure, including congestive hepatomegaly, nausea, and bilateral lower limb edema. She reported a 1-month history of worsening exertional intolerance. During hospitalization for preoperative evaluation, she developed acute pulmonary edema with profound cardiogenic shock requiring inotropic and vasopressor support.

## Past Medical History

The patient had a history of rheumatic mitral valve disease with severe mitral regurgitation, for which she had undergone mitral valve replacement 15 years prior. A #27 Carpentier-Edwards bioprosthetic valve was implanted at that time, given her childbearing age. She had no history of thromboembolic events, infective endocarditis, or previous percutaneous interventions.

## Differential Diagnosis

The differential diagnoses for her acute decompensated heart failure included the following:

• degeneration and stenosis of the bioprosthetic mitral valve,
• bioprosthetic valve thrombosis,
• infective endocarditis with obstruction of the prosthetic leaflets,
• severe pulmonary hypertension secondary to left-sided valve disease,
• acute coronary syndrome precipitating heart failure.

Comprehensive echocardiography ruled out thrombus, vegetations, and significant new mitral regurgitation, supporting bioprosthetic structural degeneration as the primary cause.

## Investigations

**Figure 1:**
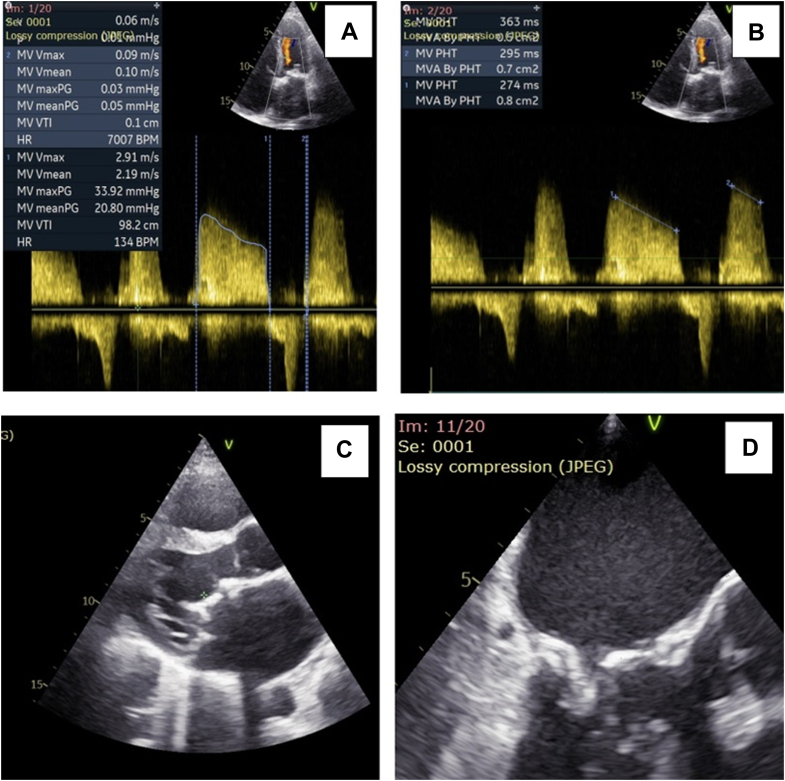
TTE and TEE Assessment of Degenerated Bioprosthetic Mitral Valve (A) Apical 4-chamber view on TTE and continuous-wave Doppler of mitral valve inflow showing a mean pressure gradient of 20 mm Hg. (B) Pressure half-time in apical 4-chamber view on TTE showing an average mitral valve area of 0.7 cm^2^. (C) TEE parasternal long-axis view showing degenerated bioprosthesis in the mitral position. (D) TEE 3-chamber view at 150° angle showing degenerated bioprosthesis in the mitral position. TEE = transesophageal echocardiography; TTE = transthoracic echocardiography.

Two-dimensional (2D) transthoracic echocardiography (TTE) demonstrated severe stenosis of the bioprosthetic mitral valve, with a mean pressure gradient of 20 mm Hg and a Doppler velocity index of 0.1; effective orifice area calculated using the continuity equation was found to be 0.32 cm^2^. The patient had a normal left ventricular ejection fraction of 65%, severe pulmonary hypertension with a pulmonary artery systolic pressure estimated at 110 mm Hg (Figure 1), along with severe tricuspid regurgitation, fair right ventricular systolic function, and a dilated inferior vena cava measuring 30 mm with <50% collapsibility.

**Figure 2:**
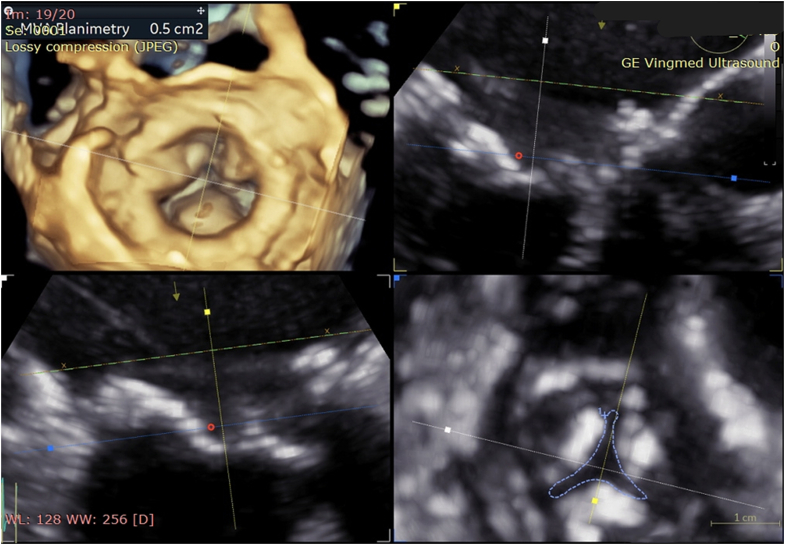
3-Dimensional Transesophageal Echocardiography Showing Severe Bioprosthetic Mitral Stenosis

Suspecting severe prosthetic valve stenosis, three-dimensional (3D) transesophageal echocardiography was performed for accurate area assessment, and 3D planimetry confirmed a valve area of 0.5 cm^2^ (Figure 2), no evidence of thrombus or vegetations, and only mild mitral regurgitation.

## Management

During her 1-week hospital stay at our facility, the patient showed initial improvement regarding her NYHA functional class and right-sided heart failure symptoms on intravenous loop diuretics, digoxin, and beta-blockers for heart rate control. She was pending transferal to cardiothoracic surgery when she experienced an episode of atrial fibrillation with rapid ventricular response, which resulted—given the severe valve stenosis—in acute pulmonary edema and cardiogenic shock. Direct-current cardioversion failed to improve her clinical condition, and redo mitral valve surgery was emergently required.

Given the patient's young age, surgical mitral valve replacement was favored over transcatheter mitral valve replacement because of the limited durability of transcatheter valves compared with metallic valves. A multidisciplinary heart team discussion concluded that the patient was at prohibitive surgical risk, with an estimated Society of Thoracic Surgeons predicted operative mortality of 45.1%; thus, we decided to perform a “bailout” transcutaneous balloon mitral valvuloplasty as a bridge to definitive surgery. The goal was to achieve adequate valve area, with a 50% increase or preferably an orifice area of >1.5 cm^2^, to ensure hemodynamic and clinical improvement rather than definitive stenosis treatment. A 25-mm balloon size was deemed optimum, as it matched the inner diameter of the #27 Carpentier-Edwards bioprosthetic after revising the bioprosthetic valve specifications.

**Figure 3:**
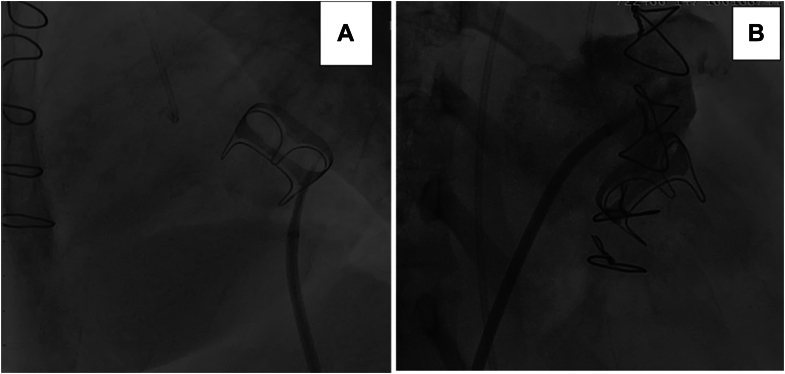
Trans-Septal Puncture During Balloon Mitral Valvuloplasty (A) Fluoroscopic image demonstrating trans-septal puncture in lateral projection with 3.5 Judkins right catheter in the aortic cusp as reference. (B) After trans-septal puncture, contrast injection via the Mullins sheath opacifies the left atrium and left atrial appendage.

**Figure 4:**
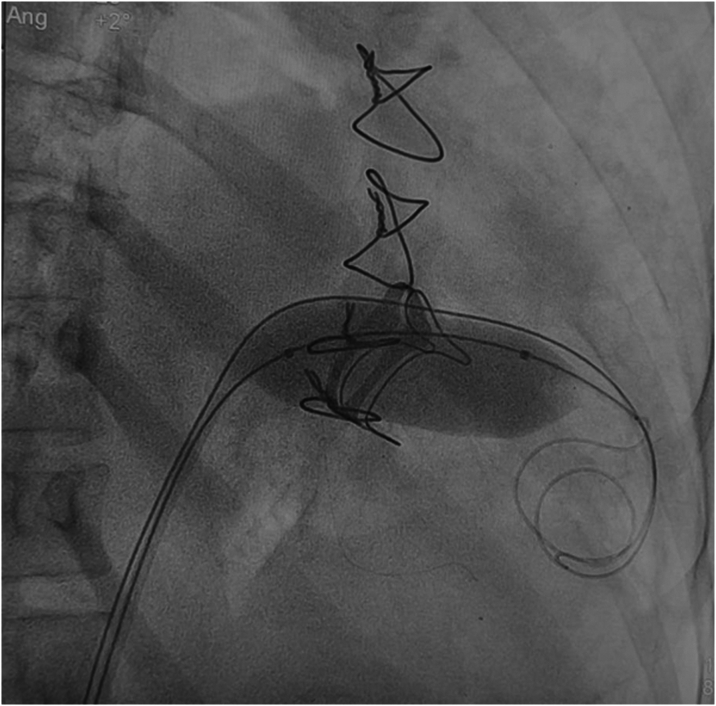
Balloon Valvuloplasty of Degenerated Bioprosthetic Mitral Valve

The procedure was performed under general anesthesia with endotracheal intubation. Invasive hemodynamic assessment revealed a left atrial pressure of 45 mm Hg, pulmonary artery systolic pressure of 120 mm Hg, and mean arterial pressure of 55 mm Hg on inotropic and vasopressor support. Trans-septal puncture was achieved using fluoroscopic and echocardiographic guidance, then over a 3.5 Judkins right catheter, 2 manually shaped Amplatz stiff wires were placed in the left ventricular apex across the prosthesis. Over one of the stiff wires, a 25 × 50 mm Crystal monorail balloon was positioned across the bioprosthetic mitral valve, while the other was used as a buddy wire for support, with manual inflation until disappearance of waist, achieving a 25-mm diameter as confirmed by fluoroscopy (Figures 3 and 4).

**Figure 5:**
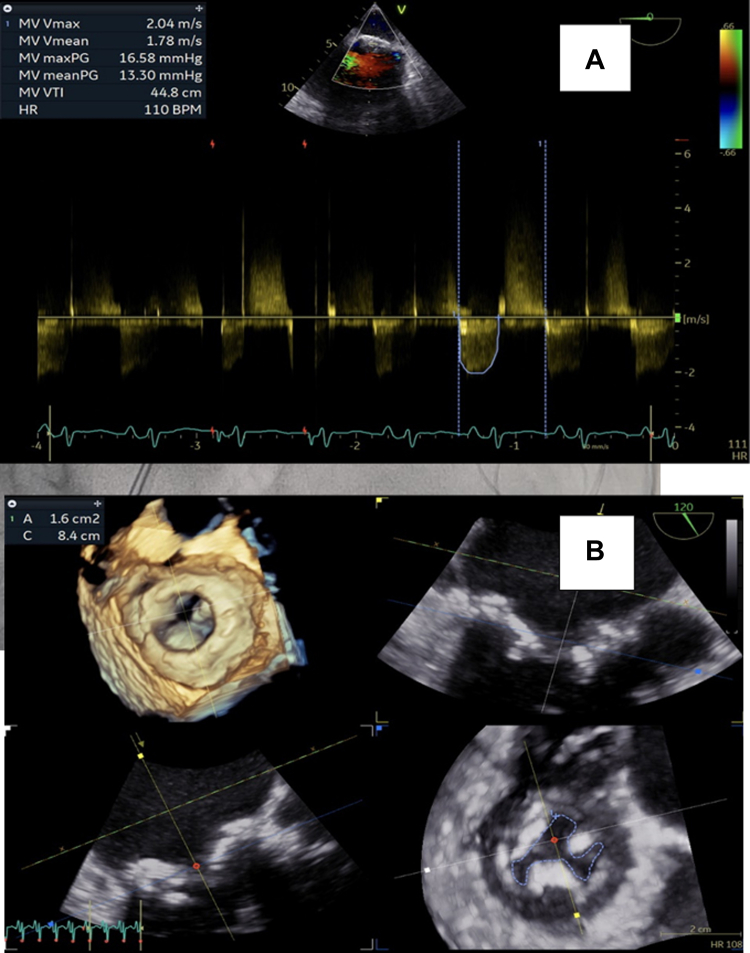
Echocardiographic Evaluation After Valvuloplasty (A) TEE 2-chamber view at 0°angle showing a mean transvalvular gradient of 13 mm Hg. (B) TEE 3D reconstruction demonstrating bioprosthesis in the mitral position after balloon valvuloplasty and orifice area measuring 1.6 cm^2^ using 3D planimetry. 3D = three-dimensional; TEE = transesophageal echocardiography.

Immediately after the valvuloplasty, there was a marked reduction in left atrial pressure from 45 to 25 mm Hg, and pulmonary artery systolic pressure decreased from 120 to 80 mm Hg. TTE showed an improved mean mitral valve gradient of 13 mm Hg (Table 1), and an increased mitral valve area to 1.6 cm^2^ with only mild mitral regurgitation, denoting successful valvuloplasty by achieving a mitral valve area of >1.5 cm^2^, which was later confirmed by 3D transesophageal echocardiography (Figure 5). The patient's clinical condition stabilized, allowing weaning of inotropic support and subsequent extubation 2 days later.

**Table 1:** Hemodynamic Parameters Before and After Percutaneous Balloon Mitral Valvuloplasty

Hemodynamic Finding	Before	After
Heart rate (beats/min)	160	120
Pressures (mm Hg)		
Mean left atrium	45	25
Pulmonary artery (systolic)	120	80
Right ventricle (systolic/end-diastolic)	120/15	80/12
Left ventricle (systolic/end-diastolic)	80/5	150/20
Aorta	70/40	130/80

## Follow-Up

After successful balloon valvuloplasty, the patient stabilized clinically, with resolution of cardiogenic shock and significant symptomatic improvement. She was maintained on intravenous diuretics and beta-blockers while monitored in the coronary care unit.

**Figure 6:**
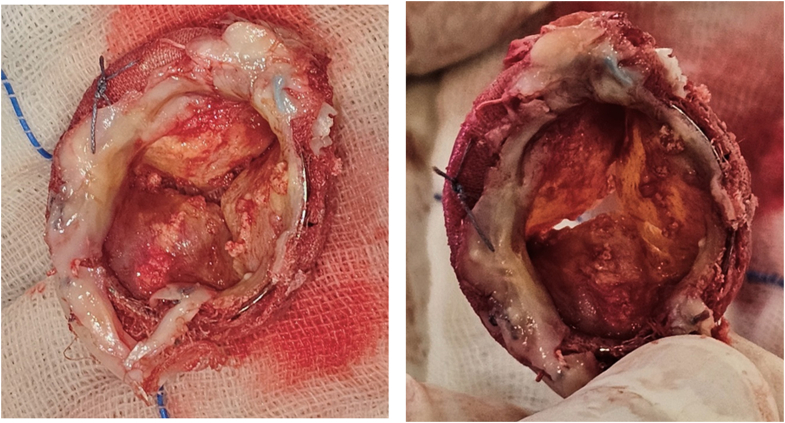
Surgically Excised Degenerated Mitral Bioprosthesis

**Figure 7:**
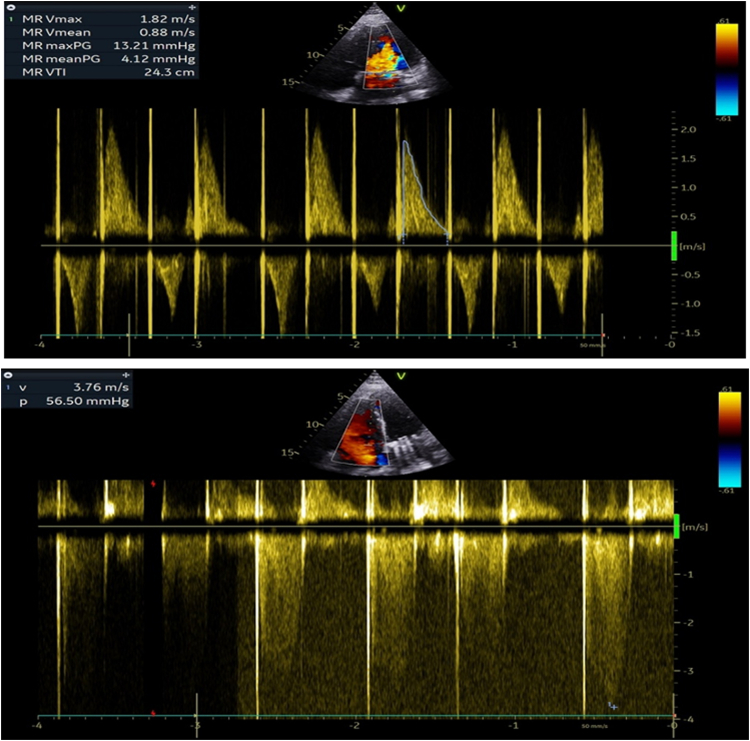
Postoperative Echocardiographic Assessment

Ten days later, she underwent successful redo mitral valve replacement with a #27 St Jude metallic prosthesis and surgical left atrial appendage ligation. Intraoperative examination confirmed severe calcific and fibrotic degeneration of the excised bioprosthetic leaflets (Figure 6). Postoperative TTE demonstrated a mean diastolic gradient of 4 mm Hg across the metallic prosthesis, with no significant transvalvular or paravalvular regurgitation. Right ventricular systolic function and dimensions were preserved, and pulmonary artery pressures showed significant improvement, with regression of tricuspid regurgitation from severe to moderate and right ventricular systolic pressure reduced to 61 mm Hg (Figure 7). The patient recovered uneventfully and was discharged home on postoperative day 7.

## Discussion

Percutaneous transcatheter balloon valvuloplasty was first performed in 1982 by Inoue et al^1^; after proof of its efficacy and safety, percutaneous mitral valvuloplasty has now become the intervention of choice in criteria-suitable cases of clinically significant native mitral valve stenosis.^2^ However, reports regarding balloon valvuloplasty for bioprosthetic valve failure in adult patients are extremely limited. A literature search identified 16 cases of balloon valvuloplasty of bioprosthesis degeneration in the mitral position (Table 2).^1^^,^^3^, ^4^, ^5^, ^6^, ^7^, ^8^ When compared with native mitral valve stenosis, bioprosthetic degeneration presents several important differences. Diagnosis can be more complex than simply assessing transvalvular gradients or pressure half-time, as these measures are often influenced by volume status and loading conditions. Moreover, 2D planimetry is frequently limited given the 3D structure of bioprosthetic valves. In this case, we relied primarily on the velocity index and 3D planimetry for accurate assessment.^9^

**Table 2:** Summary of Reported Cases of Balloon Valvuloplasty for Degenerated Mitral Bioprostheses

First Author	Age (y)/Sex	Valve Age (y)	Preprocedural PG (mm Hg)	Postprocedural PG (mm Hg)	Balloon Type	Size (mm)	Complication
Calvo et al^3^	29/M	10	15	11	Trefoil	25	Mild MR
Calvo et al^3^	37/F	3	13	3	Trefoil	25	None
Arie et al	62/F	13	13	8	Mansfield	20	None
Cox et al^5^	53/F	13	11	6	Cook	23	Mild MR restenosis
Fernandez et al^6^	62/F	10	22	6	Mansfield	20	None
Spellburg et al^7^	64/F	8	30	10	Mansfield	18	Atrial shunt
Babic et al	34/M	3	29	9	Mansfield	25	None
Orbe et al	N/A	10	15	11	Trefoil	25	Severe MR
Orbe et al	N/A	3	13	3	Trefoil	25	None
Orbe et al	N/A	7	16	5	Monoballoon	25	None
Lip et al	29/F	6	12	4	Inoue	28	None
Ludman et al	54/F	9	N/A	N/A	Inoue	N/A	None
Hurst et al	78/M	1.5	14	11	Inoue	26	None
Bekeredjian et al	86/F	9	9	5	Inoue	26	None
Hamatani et al^4^	62/F	9	30	19	Inoue	20	None
Sleiman et al^8^	88/F	7	17	8	Maxi LD	20	None

Arie S, Arato Gonçalves MT, et al. Balloon dilatation of a stenotic mitral bioprosthesis. *Am Heart J*. 1989;117:201–202. Babic UU, Grujicic S, Vucinic M. Balloon valvoplasty of mitral bioprosthesis. *Int J Cardiol*. 1991;30(2):230-232. Orbe LC, Sobrino N, Maté I, et al. Effectiveness of balloon percutaneous valvuloplasty for stenotic bioprosthetic valves in different positions. *Am J Cardiol*. 1991;68(17):1719-1721. Lip GY, Wasfi M, Halim M, Singh H. Percutaneous balloon valvuloplasty of stenosed mitral bioprosthesis. *Int J Cardiol*. 1997;59(1):97-100. Ludman P, Pitt M. Inoue balloon dilatation of a mitral valve bioprosthesis. *Heart*. 1999;81(3):320. Hurst FP, Caravalho J, Wisenbaugh TW. Prosthetic mitral valvuloplasty. *Catheter Cardiovasc Interv Off J Soc Card Angiogr Interv*. 2004;63(4):503-506. Bekeredjian R, Katus HA, Rottbauer W. Valvuloplasty of a stenosed mitral valve bioprothesis. *J Invasive Cardiol*. 2010;22(6):E97-98.

MR = mitral regurgitation; N/A = not available; PG = pressure gradient.

The mechanism of percutaneous balloon valvuloplasty also differs between native and bioprosthetic valves. In native mitral stenosis—particularly of rheumatic origin—the balloon acts by splitting fused commissures and severing fibrotic tissue. In contrast, bioprosthetic degeneration is primarily due to leaflet calcification rather than commissural fusion. Thus, the goal of balloon dilation in this context is to fracture calcific plaques and increase leaflet pliability. However, this approach carries the risk of balloon-induced leaflet tearing or cusp perforation.^10^

With the advent of transcatheter mitral valve replacement, transcutaneous valve-in-valve implantation as a treatment for bioprosthesis degeneration is being applied more frequently.^2^^,^^8^ Transcatheter valve-in-valve implantation has a Class IIa indication for bioprosthetic mitral valve degeneration in patients at high to prohibited surgical risk according to the 2021 guidelines of the European Society of Cardiology/European Association for Cardio-Thoracic Surgery.^2^ However, given anatomic, financial, and reimbursement issues, as well as durability considerations in young patients—as in our case—not all patients qualify for this procedure, and percutaneous balloon valvuloplasty is still an option, with symptomatic and hemodynamic benefit. More evidence is required to establish a solid recommendation for balloon valvuloplasty in the case of bioprosthetic mitral valve degeneration and stenosis.

## Conclusions

This case shows that percutaneous balloon valvuloplasty can serve as a life-saving bridge therapy in patients with severe bioprosthetic mitral valve stenosis who present with cardiogenic shock and are deemed unsuitable for immediate surgery or transcatheter valve-in-valve implantation. Although not a definitive solution, balloon valvuloplasty may stabilize such high-risk patients, allowing for subsequent definitive surgical treatment with improved outcomes.

**Take-Home Message**

• Balloon valvuloplasty can serve a very specific role in highly selected patients with bioprosthetic mitral valve stenosis who are at high risk for redo mitral valve replacement and valve-in-valve procedure.

**Visual Summary:** Case Timeline

Time	Events
Day 1	A 44-year-old woman presented with NYHA functional class III symptoms and decompensated systolic congestive heart failure that started 1 month earlier, requiring emergency admission to the cardiac care unit. TTE showed severe bioprosthetic mitral valve stenosis, with a mean pressure gradient of 20 mm Hg and an estimated mitral valve area of 0.7 cm^2^ by pressure half-time. Severe pulmonary hypertension was noted with a pulmonary artery systolic pressure estimated at 110 mm Hg, along with severe tricuspid regurgitation.
Day 2	Three-dimensional transesophageal echocardiogram confirmed preliminary TTE findings, and planimetry showed a valve area of 0.5 cm^2^, no evidence of thrombus or vegetations, and only mild mitral regurgitation.
Day 5	The patient experienced acute pulmonary edema and cardiogenic shock; the multidisciplinary team concluded she was at prohibitive surgical risk. She underwent balloon bioprosthetic mitral valvuloplasty, after which there was a marked reduction in left atrial pressure from 45 to 25 mm Hg, and pulmonary artery systolic pressure decreased from 120 to 80 mm Hg. TTE showed an improved mean mitral valve gradient of 13 mm Hg and an increased mitral valve area to 1.6 cm^2^, with only mild mitral regurgitation.
Day 7	The patient improved clinically, was weaned from intravenous vasopressors and inotropes, and was extubated. She was kept under monitor in the cardiac care unit, receiving intravenous diuretics and beta-blockers for rate control as preparation for surgical valve replacement
Day 15	The patient underwent successful redo mitral valve replacement with a #27 St Jude metallic prosthesis and surgical left atrial appendage ligation.
Day 18	Postoperative TTE demonstrated a mean diastolic gradient of 4 mm Hg across the metallic prosthesis, with no significant transvalvular or paravalvular regurgitation. Right ventricular systolic function was preserved, and pulmonary artery pressures showed significant improvement.
Day 25	The patient recovered uneventfully and was discharged home

TTE = transthoracic echocardiography.

## Uncited Table

Table 3.

## Funding Support and Author Disclosures

The authors have reported that they have no relationships relevant to the contents of this paper to disclose.
